# Diagnosis and surgical treatment of multiple endocrine neoplasia type 2A

**DOI:** 10.1186/1477-7819-12-8

**Published:** 2014-01-09

**Authors:** Kun-Long Tang, Yi Lin, Li-Ming Li

**Affiliations:** 1 Department of Urology, General Hospital of Tianjin Medical University, Tianjin 300052, P.R. China

**Keywords:** Diagnosis, Multiple endocrine neoplasia type 2A, Surgical excision, Treatment

## Abstract

**Background:**

This study aims to introduce the diagnosis and surgical treatment of the rare disease multiple endocrine neoplasia type 2A (MEN 2A).

**Methods:**

Thirteen cases of MEN 2A were diagnosed as medullary thyroid carcinoma (MTC) and pheochromocytoma by biochemical tests and imaging examination. They were treated by bilateral adrenal tumor excision or laparoscopic surgery.

**Results:**

Nine patients were treated by bilateral adrenal tumor excision and the remaining four were treated by laparoscopic surgery for pheochromocytoma. Ten patients were treated by total thyroidectomy and bilateral lymph nodes dissection and the remaining three were treated by unilateral thyroidectomy for MTC. Up to now, three patients have died of MTC distant metastasis.

**Conclusions:**

We confirmed that MEN 2A can be diagnosed by biochemical tests and imaging examination when genetic testing is not available. Surgical excision is the predominant way to treat MEN 2A; pheochromocytoma should be excised at first when pheochromocytoma and MTC occur simultaneously.

## Background

Multiple endocrine neoplasia type 2 (MEN 2) is an inherited syndrome which affects many endocrine glands. Generally, MEN 2 is divided into three subtypes: MEN 2A, MEN 2B, and familial medullary thyroid carcinoma [1]. MEN 2A, also known as Sipple syndrome, is a very rare disease. More than 80% of MEN 2 cases are MEN 2A and its prevalence is approximately 1 in 25,000 [2,3]. Genetic testing is currently the main method of MEN 2A diagnosis in developed countries. However, it has not yet been extensively spread in developing countries because of economic and technical limitations. Therefore, MEN 2A is normally diagnosed by biochemical tests and imaging examinations [4,5]. Surgical excision is the predominant treatment of MEN 2A. The present study aims to describe this disease and to improve people’s awareness about the diagnosis and treatment of MEN 2A.

## Methods

### Ethical statement

Any experimental research that is reported in the manuscript have been performed with the approval of Research Ethics Committee of General Hospital of Tianjin Medical University. Research carried out on humans was in compliance with the Helsinki Declaration.

### Patients

Thirteen MEN 2A patients (2 male and 11 female; mean age, 37.9 years; mean course of disease, 1.8 years) were admitted to our hospital from 1988 to 2011. Ten of 13 patients were diagnosed as MEN 2A after presenting with paroxysmal hypertension, cardiopalmus, and dizziness, and the remaining 3 were diagnosed following a physical examination. MTC occurred earlier than pheochromocytoma in 8 cases; MTC and pheochromocytoma occurred simultaneously in 5 cases. No patients showed hyperparathyroidism.

### Diagnosis and treatment

Twenty-hour urinary vanillylmandelic acid (VMA) was tested in all patients. Its levels were significantly elevated in 10 patients and the highest was 950 μmol/L (normal 24–70 μmol/L). Serum calcitonin levels were highly increased in 7 cases and the highest was 3,470 μmol/L (normal 28–133 μmol/L). Parathyroid hormone levels were normal in 10 patients.

**Figure 1:**
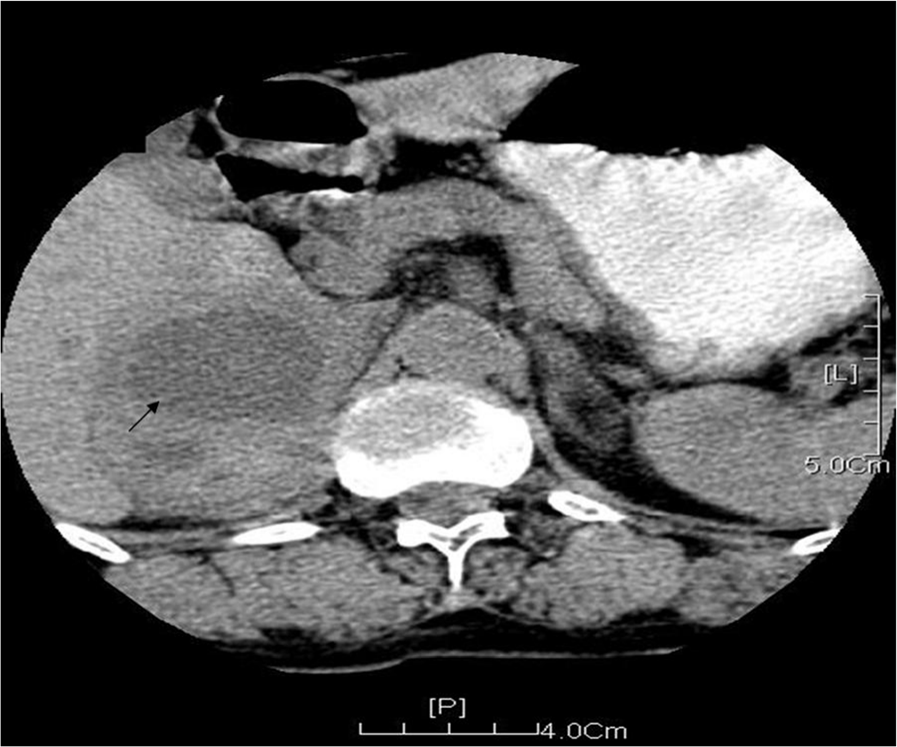
Plain CT scanning of the adrenal gland shows a uneven mass (Black arrow, about 6.2 × 6.7 × 7.8 cm) with intact capsule and punctate calcifications located at the right adrenal gland, which shares a unclear boundary with margo interior hepatics.

**Figure 2:**
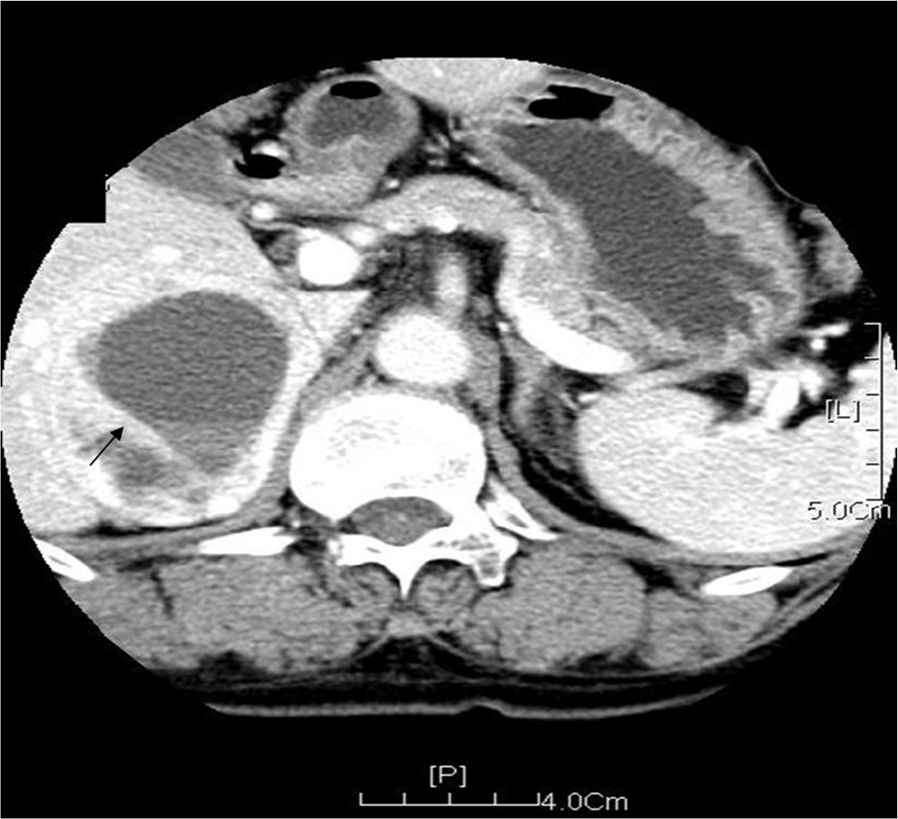
**Enhanced CT scanning of the adrenal gland shows a round-like cystic solid mass located at the right adrenal gland (Black arrow).** Inside of the mass, multiple punctate and dense shadows are visible. The boundary of the mass is manifested as inhomogeneous enhancement. Mass center shows a lamellar non-enhanced area and separation.

**Figure 3:**
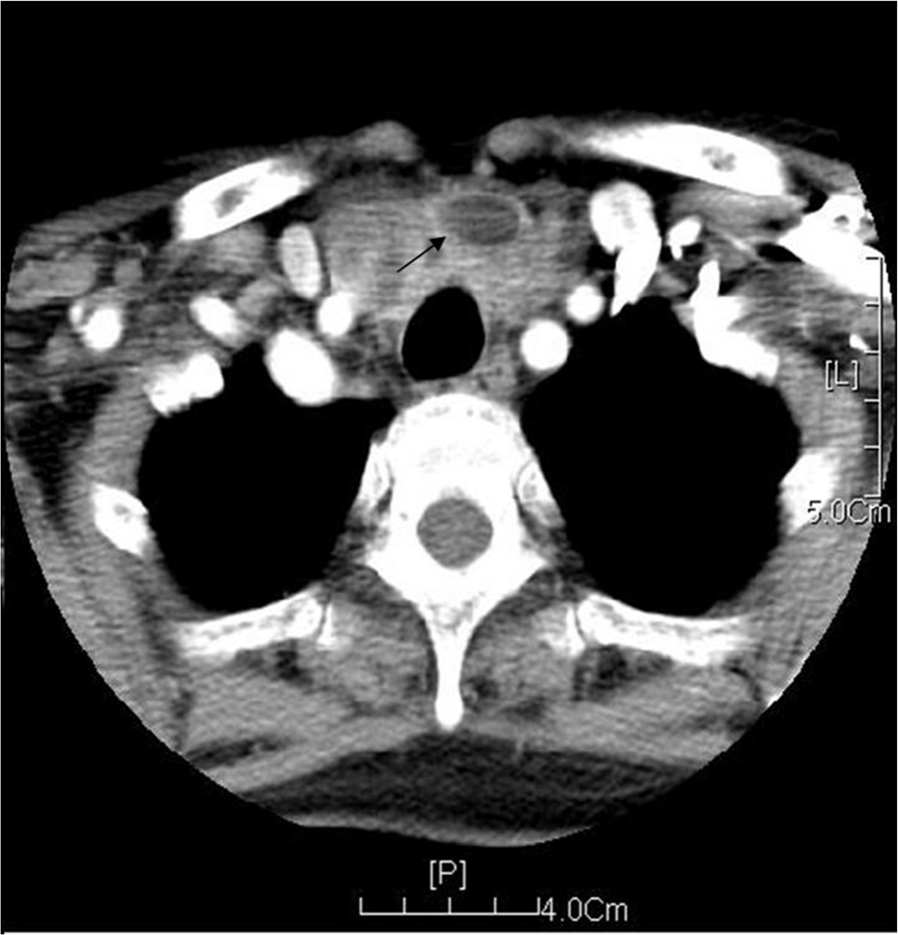
**Enhanced CT scanning of the thyroid gland shows a cystic-solid low density mass, which is located underneath lateral lobes of the thyroid gland and manifested as inhomogeneous enhancement (Black arrow).** Inside of the mass, a lamellar non-enhanced area is visible. The mass is projected into the gaps of surrounding vessels. The cervical part shows no obvious enlarged lymph nodes.

Adrenal B-mode ultrasonography was performed in 13 patients. We found 9 cases of bilateral lesions, 1 case of left side lesion, 2 cases of right side lesions, and 1 case of ectopic pheochromocytoma located at one side of the abdominal aorta. Computed tomography (CT) of adrenal gland was performed in 10 patients. The images revealed uneven mass with intact capsules (Figure 1) and they were more obvious on enhanced CT images (Figure 2). Magnetic resonance imaging (MRI) was performed in 4 patients and the images showed even or uneven masses with intact capsules. Thyroid B-mode ultrasonography was performed in 13 patients and revealed low echogenic regions with uneven echo. Thyroid CT was performed in 10 patients and showed inhomogeneous low density images (Figure 3).

### Surgical and medical treatment

**Figure 4:**
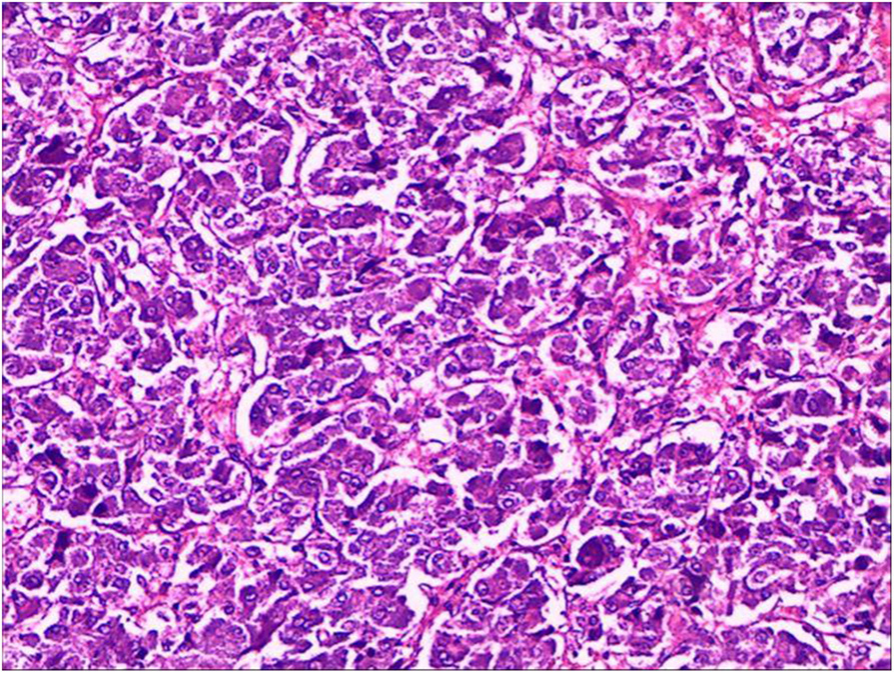
**Pathologically diagnosed adrenal pheochromocytoma (HE × 400) shows a wide variety of tissue structure and cell morphology.** Tumor tissue is separated into lobulated parts by fibrous bands come from envelope. Tumor cells (most are polygonal, few are fusiform and prismatical) are mostly arranged in cords, nests, and pieces, which are separated by thin-walled blood sinuses and fibrous tissues rich in blood vessels. Tumor cells show unclear boundaries and have lots of cytoplasm as well as inhomogeneous particles (slightly basophilic or amphophilic). Tumor cell nucleus is round or oval, which has obvious nucleolus and little karyokinesis.

All patients were treated with surgical operation; 9 patients received adrenal open operation and the remaining 4 received peritoneoscope minimally invasive treatment. All excised masses had intact capsules and were proven to be pheochromocytoma (Figure 4). Radical thyroidectomy and neck lymph node dissection were performed in 10 patients. Three patients were treated by unilateral thyroidectomy and the remaining thyroids were excised in 5, 7, and 10 years after initial thyroidectomy because of their MTC recurrence. Pheochromocytoma was excised in advance and thyroidectomy was performed 2 weeks later in the 5 cases with MTC and pheochromocytoma occurred simultaneously. All patients were treated with oral thyroxin tablet after surgery and 6 patients took prednisone together. Both medicines were taken perpetually.

Patients were followed-up after surgery for tumor recurrence. The following items were included in the follow-up: family illness history, biochemical tests (serum calcitonin, cortisol, thyroid hormone, urinary VMA), and imaging examination (ultrasound, CT).

## Results

Patients’ primary symptoms related to pheochromocytoma disappeared after operation. Ten cases had normal blood pressure and the remaining 3 had hypertension. VMA of one patient was higher than normal after surgery and one adrenal lump was discovered at the opposite side of surgical position in this patient 3 years later. It was subsequently excised and proven to be pheochromocytoma. Ten patients are still alive and 3 patients died of MTC distant metastasis at the time of writing this manuscript.

## Discussion

MEN 2A is an autosomal dominant syndrome involving multiple endocrine glands. It is characterized by MTC, pheochromocytoma, and hyperparathyroidism. MTC occurs in nearly all MEN 2A patients and is generally the first manifestation of MEN2A, whereas pheochromocytoma and hyperparathyroidism occurs in 30%–50% and 20% of MEN 2A patients, respectively [6,7]. Hyperparathyroidism is mainly due to diffuse hyperplasia of the parathyroid glands and not to adenoma. The syndrome affects all ages and both sexes with equal frequency. However, all of our 13 patients presented with MTC and pheochromocytoma without hyperparathyroidism, there were more female patients (n = 11) than male patients (n = 2). These deviations may be related to the small sample size.

MEN 2A is associated with germ line mutation of *RET* proto-oncogene [8]. Genetic testing can detect missense mutation of *RET* proto-oncogene, which makes it a reliable method to diagnosis MEN 2A [9]. However, we could not perform this test because of technical limitations. MTC tumor cells can produce many biochemical substances, such as calcitonin, adrenocorticotropic hormone, histamine, and carcinoembryonic antigen. Calcitonin is a specific tumor marker which is critical in the diagnosis of MTC as well as in determining whether the tumor has been completely excised and in monitoring of tumor recurrence [10-12]. Generally, serum calcitonin levels of MTC patients are higher than normal [13]. Pheochromocytoma is an adrenal gland tumor that produces excess adrenaline; VMA is a terminal metabolite of adrenaline. Some researchers have demonstrated that urinary VMA is specific and sensitive to the diagnosis of pheochromocytoma [14,15]. The detection of serum calcium and parathyroid hormone can improve the detection rate of MEN 2A [16].

Imaging examinations such as B-mode ultrasonography, MRI, and CT, play a vital role in initial tumor localization and the management of MEN 2A [17]. Specifically, B-mode ultrasonography is the preferred method of diagnosis and localizing MTC and coexisting parathyroid hyperplasia or adenoma. CT examination is the first choice for localizing pheochromocytoma. Although MRI has inferior localizing capability when compared with CT and B-mode ultrasonography, it is very helpful to explore tumor invasion as well as the relationship between the tumor and the great vessels.

Surgical excision is the main mode of management of MEN 2A patients. Pheochromocytoma should be excised initially in those patients with simultaneous MTC and pheochromocytoma. Otherwise, surgeries may lead to the onset of fatal hypertension [18]. Moreover, preoperative preparation should be done thoroughly by using 2 weeks of specific α-receptor blocker and 1 week of intravenous infusion. In MEN 2A patients, most pheochromocytomas are bilateral in nature, and should be preferably removed by bilateral tumor excision. Abdominal incision or curved incision can achieve better surgical exposure during operation [19]. In our study, 9 of 13 patients were diagnosed with bilateral pheochromocytoma and all of them underwent bilateral tumor excision. However, it should be noted that, in order to prevent adrenal crisis, prednisone hormone replacement therapy should be performed during and after surgery. Laparoscopy, a newly developed minimally invasive approach, which can lead to minor intraoperative injury and rapid postoperative recovery, was performed in 4 cases of unilateral pheochromocytoma MEN 2A patients and their pheochromocytoma was successfully removed.

MEN 2A-related MTC is susceptible to cervical lymph node metastasis and hematogeneous spread; therefore, total thyroidectomy with bilateral lymph node dissection should be carried out and the parathyroid glands should be explored at the same time even in unilateral MTC [20]. As for our patients, 10 patients received total thyroidectomy and bilateral lymph node dissection, the remaining 3 were treated by unilateral thyroidectomy and their remained thyroid was separately excised 5, 7, and 10 years after first operation because of MTC recurrence. Because the thyroid surgery of these 3 patients was performed in a junior hospital, the neck dissection was not performed at the same time due to the limited medical level.

Prognosis of MEN 2A is primarily dependent upon the staging of MTC [21]. Therefore, it is very important to monitor serum calcitonin regularly. It is recommended that serum calcitonin should be examined 1, 3, 6, and 12 months after initial thyroidectomy and twice a year thereafter. B-mode ultrasonography or CT examination of the neck should be performed once a year to find the recurrence and metastasis of MTC and the prognosis of MEN 2A timely. Three patients were diagnosed with MTC recurrence by serum calcitonin test during follow-up.

## Conclusions

In conclusion, MEN 2A can be diagnosed by biochemical tests and imaging examination when genetic testing is not available. Surgical excision is the predominant way to treat MEN 2A and pheochromocytoma should be excised at first when pheochromocytoma and MTC occur simultaneously.

### Consent

Written informed consent was obtained from the patient for the publication of this report and any accompanying images.

## Abbreviations

CT: Computed tomography; MEN2: Multiple endocrine neoplasia type 2; MRI: Magnetic resonance imaging; MTC: Medullary thyroid carcinoma; VMA: Vanillylmandelic acid.

## Competing interests

The authors declare that they have no competing interests.

## Authors' contributions

KLT designed the study and drafted the manuscript, YL and LML carried out the operation and collected the data. All authors read and approved the final manuscript.
